# A large Middle Devonian eubrachythoracid ‘placoderm’ (Arthrodira) jaw from northern Gondwana

**DOI:** 10.1186/s13358-020-00212-w

**Published:** 2021-01-14

**Authors:** Melina Jobbins, Martin Rücklin, Thodoris Argyriou, Christian Klug

**Affiliations:** 1 Paläontologisches Institut und Museum, Universität Zürich, Karl-Schmid-Strasse 4, 8006 Zurich, Switzerland; 2 Naturalis Biodiversity Center, Leiden, The Netherlands; 3 UMR 7207 (MNHN – Sorbonne Université – CNRS) Centre de Recherche en Paléontologie, Muséum National D’Histoire Naturelle, 57 rue Cuvier, 75231 Paris cedex 05, France

**Keywords:** Arthrodira, Dentition, Food web, Givetian, Maïder basin, Palaeoecology

## Abstract

For the understanding of the evolution of jawed vertebrates and jaws and teeth, ‘placoderms’ are crucial as they exhibit an impressive morphological disparity associated with the early stages of this process. The Devonian of Morocco is famous for its rich occurrences of arthrodire ‘placoderms’. While Late Devonian strata are rich in arthrodire remains, they are less common in older strata. Here, we describe a large tooth-bearing jaw element of *Leptodontichthys ziregensis* gen. et sp. nov., an eubrachythoracid arthrodire from the Middle Devonian of Morocco. This species is based on a large posterior superognathal with a strong dentition. The jawbone displays features considered synapomorphies of Late Devonian eubrachythoracid arthrodires, with one posterior and one lateral row of conical teeth oriented postero-lingually. μCT-images reveal internal structures including pulp cavities and dentinous tissues. The posterior orientation of the teeth and the traces of a putative occlusal contact on the lingual side of the bone imply that these teeth were hardly used for feeding. Similar to *Compagopiscis* and *Plourdosteus*, functional teeth were possibly present during an earlier developmental stage and have been worn entirely. The morphological features of the jaw element suggest a close relationship with plourdosteids. Its size implies that the animal was rather large.

**ZooBank LSID:** urn:lsid:zoobank.org:pub:B707FB56-74A8-42C2-8BA0-D5BE1F32AB4A.

## Introduction

‘Placoderms’ are considered as a paraphyletic grade on the stem of jawed vertebrates by many researchers (Brazeau 2009; Brazeau and Friedman 2015; Zhu et al. 2013), although there have been recently suggestions of monophyly (King et al. 2016) and monophyly of at least some ‘placoderms’ (Vaškaninová et al. 2020). Characterised by their well-developed head and thoracic dermal bony armor, ‘placoderms’ are known from the Silurian (Wang 1991; Zhu et al. 2016) to the end of the Devonian (Brazeau and Friedman 2015). Their phylogenetic position as successive sister groups of crown-group jawed vertebrates (Brazeau and Friedman 2015) makes them important to reconstruct character evolution in early vertebrates. In particular, remarkable advances in the understanding of the origin and homology of crown gnathostome cheek and jaw bones (Zhu et al. 2013, 2016), the origin of teeth on jaws (Smith and Johanson 2003a; Rücklin et al. 2012; Vaškaninová et al. 2020), the origin of endochondral bone (Brazeau et al. 2020), and the origin of internal fertilization and live birth (Long et al. 2008, 2009; Trinajstic et al. 2015) have been made in the past two decades through the study of ‘placoderm’ fossils. These morphological innovations possibly fuelled the further diversification of vertebrates, the occupation of the according new ecological niches, and the overall evolutionary success of jawed vertebrates. The great diversity of forms in the Devonian reflects diverse modes of life adopted by ‘placoderms’ including quite different habitats and feeding strategies (Miles 1969; Vaškaninová and Kraft 2014; Trinajstic and Roelofs 2018; Coatham et al. 2020). Devonian ‘placoderms’ show a number of adaptations from active demersal to pelagic swimming, with some arthrodire species considered to have been apex predators in Late Devonian ecosystems (Anderson and Westneat 2006).

A main feature that drove the diversification in diet and food acquisition is the development of jaws with bony dermal gnathal elements that may be covered in semidentine, a histological structure characterized by unipolar cell lacunae. This tissue type is a putative synapomorphy of ‘placoderms’ (Young 2010). Due to their conspicuously developed exoskeleton and wide geographic range, arthrodires are the most common and best documented ‘placoderms’. Considered as a sister group to Ptyctodontida and the Silurian maxillate ‘placoderm’ *Quilinyu* (Zhu et al. 2016), arthrodires occur globally. Exceptionally preserved specimens from Konservat-Lagerstätten, such as the Gogo Formation in Australia (e.g. Dennis and Miles 1980; Dennis-Bryan 1987; Dennis-Bryan and Miles 1983; Gardiner and Miles 1994; Long 1995; Young 2003b; Long and Trinajstic 2010) or various Moroccan sites (Lehman 1956, 1964; Lelièvre 1984a, b, 1995; Rücklin 2011; Rücklin and Clément 2017), greatly improved our knowledge of their palaeobiology.

Their upper jaws comprise of two pairs of superognathals, the anterior and posterior ones, while the lower jaw is formed by the inferognathal situated on the Meckel’s cartilage, which is ossified anteriorly as the mentomeckelian and posteriorly as the articular. Dentitions and jaw bones of Devonian arthrodires have featured in recent discussions on tracing the plesiomorphic condition of jaws (Zhu et al. 2016) and the origin of teeth in the first jawed vertebrates (Donoghue and Rücklin 2014; Johanson and Smith 2005; Rücklin et al. 2012; Smith and Johanson 2003a, b). The most popular ongoing debate pertains to the homology of teeth in ‘placoderms’ and crown-gnathostomes. Several researchers argued that these were merely tubercles that resemble teeth and did not develop from a dental lamina, assumed to be a key character for teeth in gnathostomes (Burrow 2003; Young 2003a). However, it is shown that patterned teeth can develop without a dental lamina (Huysseune and Witten 2008; Vandenplas et al. 2014). Several studies revealed that, despite the lack of some processes such as tooth resorption, ‘placoderms’ exhibit structures homologous to crown gnathostome teeth, which include a dentinous tissue, a pulp cavity and a successional development out of bite (Donoghue and Rücklin 2014; Johanson and Smith 2005; Rücklin et al. 2012, 2014; Smith and Johanson 2003a, b; Vaškaninová et al. 2020). These dental structures grow through successive addition and form rows, as in the arthrodire *Compagopiscis* (Donoghue and Rücklin 2014; Rücklin et al. 2012) and also inferred based on gross morphology for the arthrodire *Plourdosteus* (Ørvig 1980). However, these studies focused mainly on Late Devonian eubrachythoracids and Early Devonian buchanosteids. Gnathal elements from older eubrachythoracids have not been analyzed so far in detail. This is even more notable for the superognathals with the only Middle Devonian eubrachythoracid records of *Coccosteus cuspidatus* from Scotland (Miles and Westoll 1968; Johanson and Smith 2005) and *Kiangyousteus yohii* from China (Zhu and Zhu 2013). Very few arthrodire dentitions have been histologically examined so far (Ørvig 1980; Rücklin et al. 2012) and these do not include *C. cuspidatus* and *K. yohii*. As a result, issues regarding the evolutionary history and putative function of these dental structures remain open.

In this article, we (1) describe a tooth-bearing posterior superognathal of a large Middle Devonian arthrodire, (2) introduce a new genus and species, (3) discuss its systematic position, (4) compare morphological details of its teeth to other early jawed vertebrates, and (5) assess its functional morphology as well as (6) its putative diet and the implied role in Middle Devonian food webs of the Maïder Basin.

## Materials and methods

### Geological setting

**Fig. 1:**
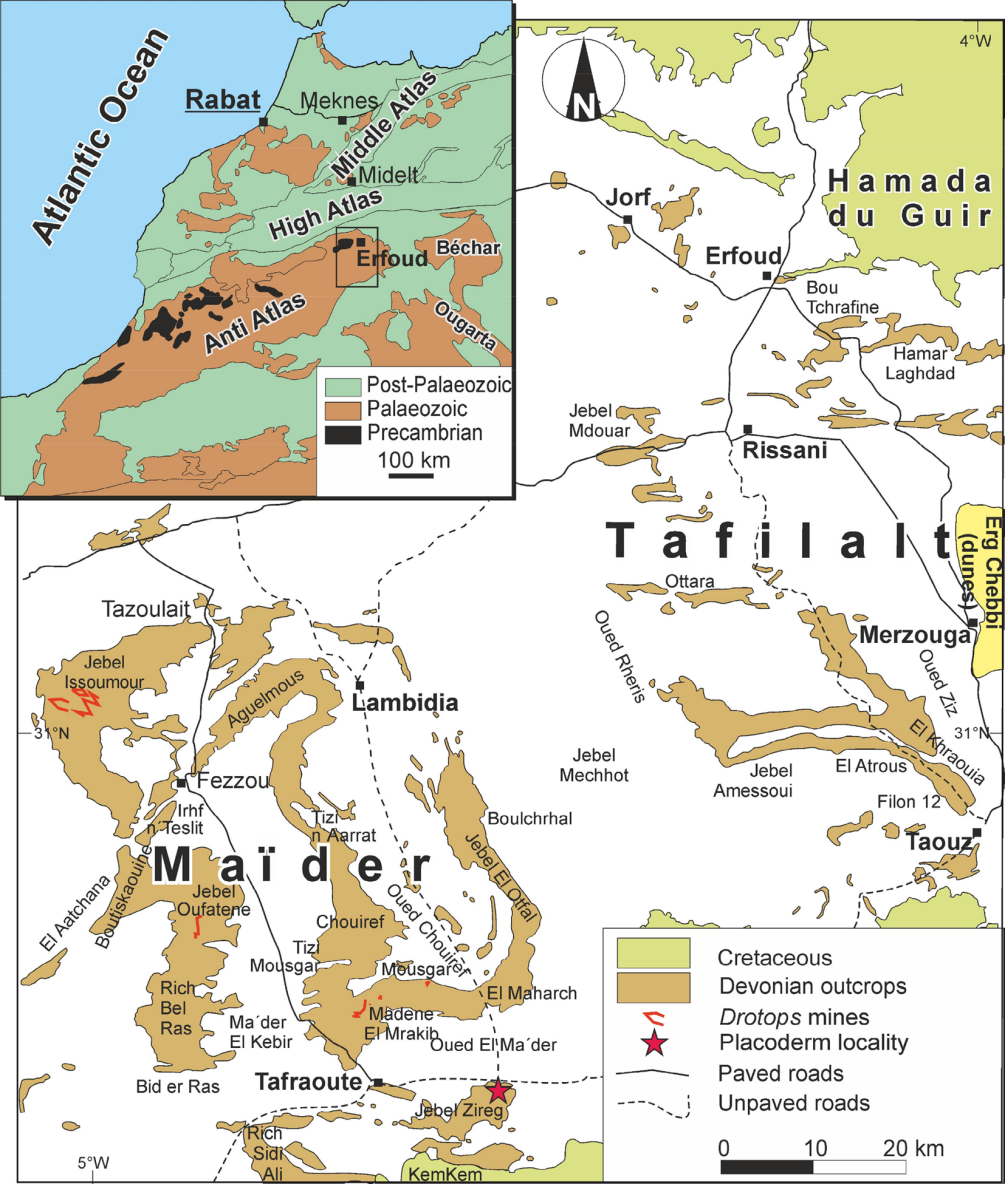
Map of the Anti-Atlas of Morocco, showing where the *Drotops* layer crops out and Jebel Zireg, where the holotype of *Leptodontichthys ziregensis* gen. et sp. nov. was found

The specimen discussed herein was found in the Moroccan eastern Anti-Atlas, where middle Palaeozoic sediments are widely exposed (Fig. 1). These sediments formed two small epicontinental basins (Wendt 1985). The western basin is commonly called Maïder Basin (Frey et al. 2018, 2020). There, the Devonian succession reaches more than 1000 m in thickness (Wendt 1985), of which Middle Devonian carbonates and argillites may measure over 500 m in the depocentre (Kaufmann 1998). In the south and west of the basin, the globally largest known genus of the trilobite family Phacopidae occurs in a short stratigraphic interval: *Drotops* comprises two species, which mainly differ in the presence of 10-mm-long spines in *D. armatus* where *D. megalomanicus* (almost 200-mm long) carries strong tubercles (Struve 1995). These strata yield abundant remains of sessile benthic organisms such as brachiopods, corals and crinoids. Very few vertebrates were described from the same layer, including some conodonts (Kaufmann 1998; Lelièvre 1995; Campbell et al 2002; Jakubowicz et al 2019), the arthrodire *Maideria falipoui* (Lelièvre 1995) and the dipnoan *Dipnotuberculus gnathodus* (Campbell et al. 2002). Now, we can add a large arthrodire to this list. Previous research using conodonts dated the locality to the early to middle Givetian (Lelièvre 1995; Kaufmann 1998; Campbell et al. 2002; Jakubowicz et al. 2019).

### Materials

The fossil studied here is a right posterior superognathal (PSG). The PSG is stored at the Palaeontological Museum collections of the University of Zurich (PIMUZ A/I 5022). Stratigraphic and geographic information is provided in the systematic part. The fin spine of a large acanthodian (*Machaeracanthus major*; PIMUZ A/I 5023) was found in the same locality.

### Methods

#### Preparation

The specimen was prepared using an alternation of chemical and physical methods. Large areas of matrix were removed with an air scribe. The more fragile areas, like the teeth, were prepared with 5% acetic acid following the methods of Toombs and Rixon (1959) and Rixon (1976). A layer of varnish made of Uhu Hart (Bolton adhesives) glue diluted with acetone was applied to fix the bone and prevent corrosion by the acid or mechanical damage during the acid preparation.

#### Photography, digital tomography and segmentation

Photographs were taken with a Nikon D2X and a Nikon AF Nikkor 35–70-mm 2.8 lens. The specimen was scanned before and after preparation using a Nikon XT H 225 ST Computed Microtomography (μCT) system at the University of Zurich. The interpretations presented here are based on a scan of the complete posterior superognathal (parameters: voxel size of 0.049 mm; 210 kV; 219 uA; 1-mm copper filter), and close-up scans of the lateral (parameters: voxel size of 0.025 mm; 200 kV; 120 uA; 1-mm copper filter) and posterior (parameters: voxel size of 0.025 mm; 210 kV; 119 uA; 1-mm copper filter) processes. The resulting tomographic volumes were segmented with Mimics v.19 software (https://www.materialise.com/en/medical/software/mimics, Materialise, Leuven, Belgium). Segmentation, 3D model reconstruction and virtual thin sections were used to visualize the anatomy of the dentition. Tomographic data and 3D models are available on Dryad (doi: 10.5061/dryad.p5hqbzkng) following the best practice guidelines for three-dimensional digital morphology data (Davies et al. 2017).

## Systematic paleontology

Placodermi McCoy, 1848.

Arthrodira Woodward, 1891.

**Eubrachythoraci** Miles, 1971

Plourdosteidae Vézina, 1990

*Leptodontichthys* gen. nov.

**Type species** *Leptodontichthys ziregensis* gen. et sp. nov.

**Etymology** Derived from the Greek words *Leptos* (Greek for slender), *odont* (declinated form of the Greek word *Odous* for tooth), and *ichthys* (Greek for fish) referring to the long and slender teeth on the posterior superognathal.

**Diagnosis** Monospecific genus, as for species.

*Leptodontichthys ziregensis* gen. et sp. nov.

**Etymology** Referring to the type locality Jebel Zireg.

**Holotype** PIMUZ A/I 5022, a right posterior superognathal.

**Locus typicus** Eastern Jebel Zireg, Maïder region, Morocco.

**Stratum typicum** *Drotops* Layer, *hemiansatus* or lower *varcus* conodont Zone, early or middle Givetian, lower Taboumakhlouf Formation, Middle Devonian.

**Material** Only the holotype.

**Diagnosis** Large arthrodire with a sub-rectangular posterior superognathal with two rows of large teeth. Teeth have a strong posterior and slight lingual orientation, with a strong upward curvature. The lateral row emerges in the middle of the bone, with five large and sharp teeth. The posterior row has smaller posteriorly oriented teeth that are nearly parallel to the occlusal plane. The biting edge is deprived of teeth. No mesial row present. Dorsal process has a ventral depression. Both ventral margins occurring before the lateral and posterior tooth rows are smooth.

### Description

The holotype PIMUZ A/I 5022 is a three-dimensionally preserved right posterior superognathal (PSG), which is approximately 108-mm long and 50-mm (52 mm with the ventral tooth of the lateral row) high. It has two rows of conical slender pointed teeth.

#### Overall shape

**Fig. 2:**
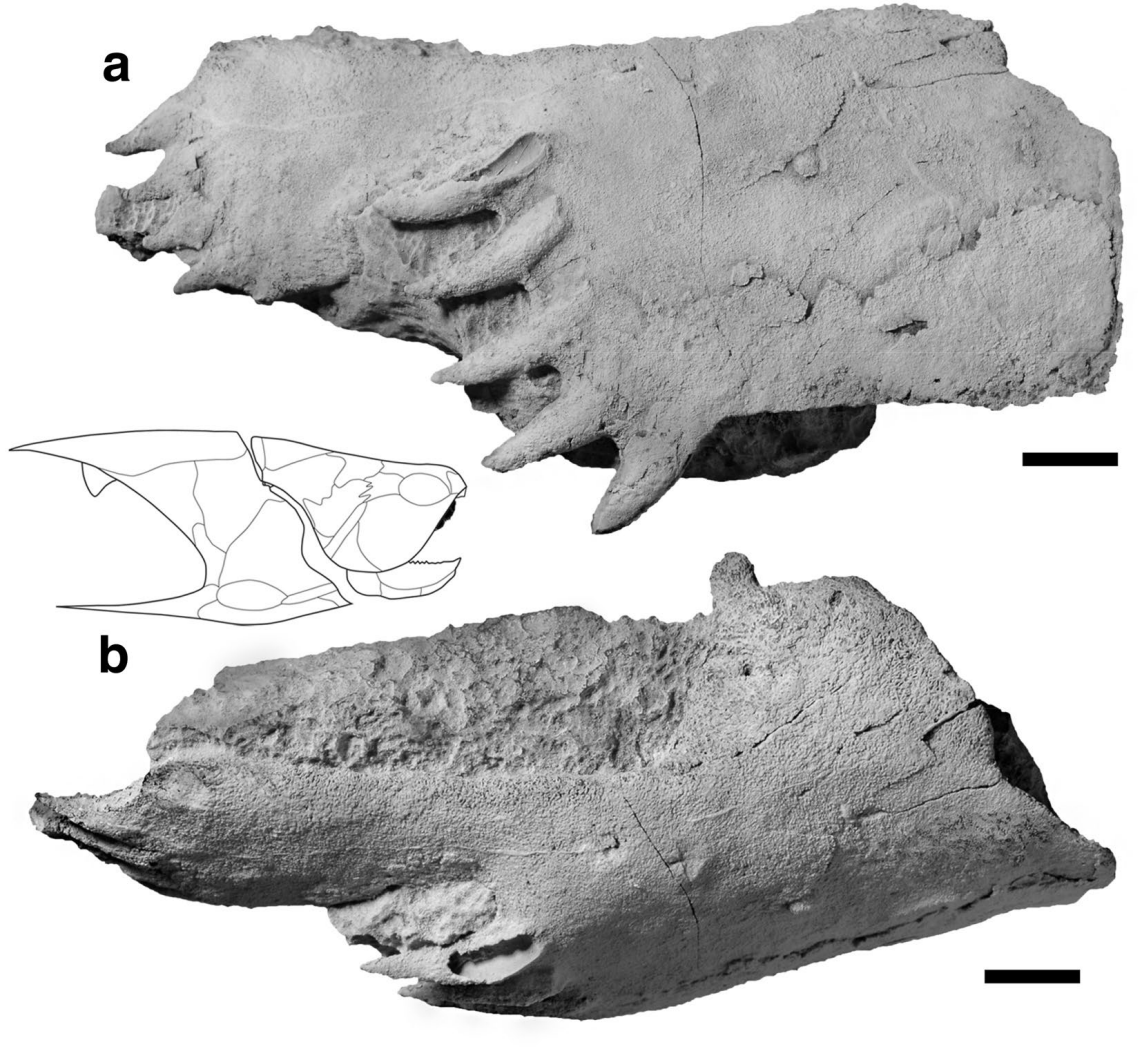
Posterior superognathal (PSG) of *Leptodontichthys ziregensis* gen. et sp. nov. (PIMUZ A/I 5022), holotype, *Drotops*-Limestone, Middle Devonian, Jebel Zireg, Morocco in labial (**a**) and dorsal view (**b**). Teeth point caudally. The PSG is highlighted in black in the reconstruction of the eubrachythoracid *Coccosteus*. Scale bar represents 10 mm

**Fig. 3:**
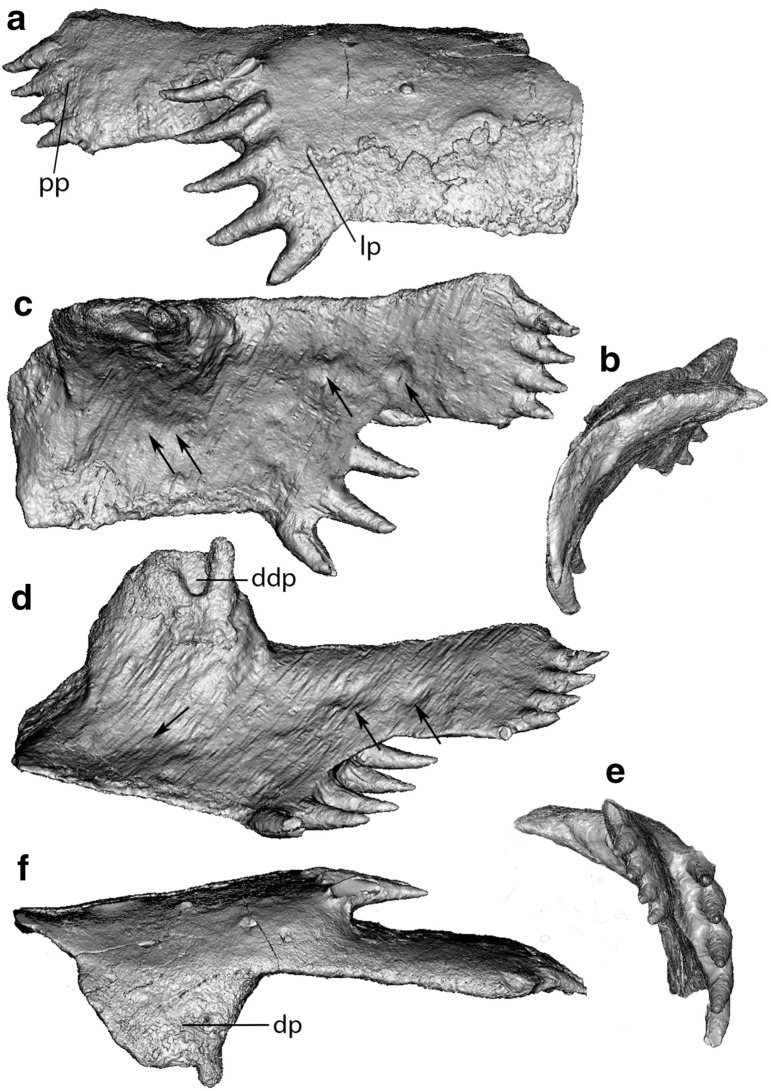
CT reconstruction of the posterior superognathal of *Leptodontichthys ziregensis* gen. et sp. nov. (PIMUZ A/I 5022) in labial (**a**), anterior (**b**), lingual (**c**), ventro-lingual (**d**), posterior (**e**) and dorsal (**f**) view. Putative occlusal marks of the inferognathal are indicated by arrows in **c** and **d**. Scale is 100 mm. dp, dorsal process; ddp, depression of the dorsal process; lp, lateral process; pp, posterior process

The PSG is sub-rectangular and dorsoventrally bent (Fig. 2). It is about twice as long as wide. The minimal height reaches 23 mm shortly anterior the posterior row of teeth, where the bone then gets a little wider (29 mm). The PSG consists of a lateral and a posterior row of teeth. The bone is 41-mm high at the lateral dental field until it reaches its maximum height at the anterior end, where a well-defined dorsal process is located. The thickness also varies along the bone, from 3 mm at the posterior margin to 10 mm on the anterodorsal margin. The dorsoventral vaulting increases dorsally towards the anterior end of the bone. The dorsal process (Fig. 2b) shows a depression on the ventral side (Fig. 3d).

#### Tooth rows and form

**Fig. 4:**
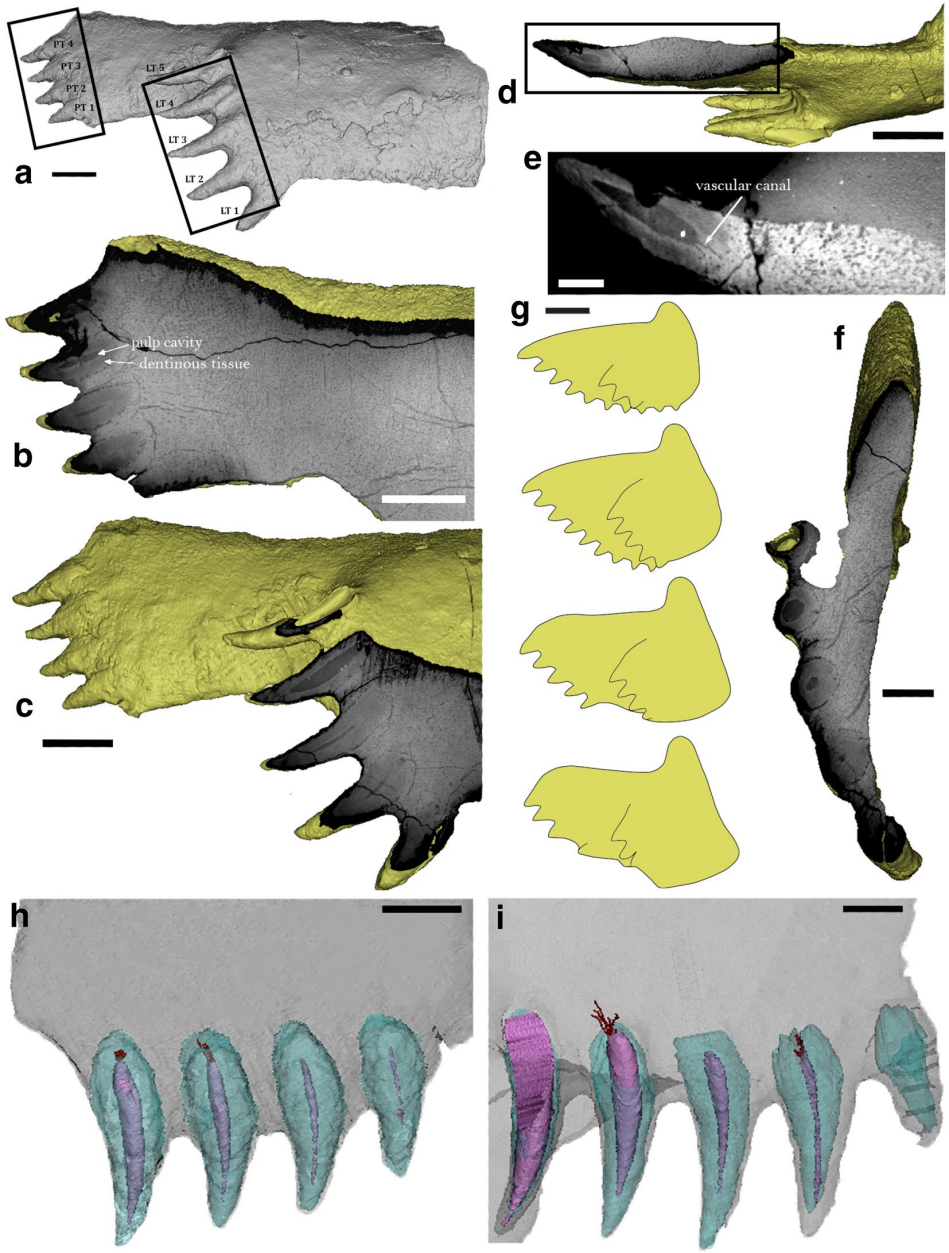
CT slices of the posterior superognathal of *Leptodontichthys ziregensis* gen. et sp. nov. (PIMUZ A/I 5022). **a** Overview in labial view. **b**–**f**, both tooth rows showing layers of dentin and the pulp cavities. **b** Parasagittal view of the posterior row and **c** of the lateral row. **d**, **e**, transverse sections (**e**: close-up). **f** Frontal section. **g** Growth development of *Plourdosteus canadensis* according to Ørvig (1980). **h** Posterior row and **i** lateral row. Both tooth rows of *L. ziregensis* gen. et sp. nov. were 3D reconstructed to show these layers and some preserved basal canals. Scale bars represent 10 mm (**a**–**d**, **g**), 2 mm (**e**), 4 mm (**f**) and 5 mm (**h**, **i**). LT, Lateral Tooth; PT, Posterior Tooth

Two rows of teeth are present with a posterior orientation (Fig. 4a). The lateral row comprises five teeth (Table 1, Lateral tooth 1–5). Their exposed lengths range from 10 mm (Lateral tooth 1) to 15 mm (Lateral tooth 5), with their widths varying between 4.2 mm (Lateral tooth 5) and 6.7 mm (Lateral tooth 1). Partially, due to erosion, the tooth base is visible in the three upper teeth (Lateral tooth 3–5). Lateral tooth 5 is broken (or eroded), thus exposing parts of its inner structure.

**Table 1:** Measurements made of the teeth of *Leptodontichthys ziregensis* gen. et sp. nov. of the lateral and posterior tooth rows are given from the most ventral (Posterior/Lateral tooth 1) to the most dorsal tooth (Posterior tooth 4 and Lateral tooth 5)

	Max exposed length	Total length	Max pulp cavity length	Max thickness tooth	Max thickness pulp cavity
Lateral tooth 1	10	14	0	6.7	0
Lateral tooth 2	12	17	14	5.6	1.1
Lateral tooth 3	12	19	13	5.2	1.8
Lateral tooth 4	12	18	14	4.8	2.5
Lateral tooth 5	15	20	18	4.2	4
Posterior tooth 1	5	10	5	3.3	0.3
Posterior tooth 2	6	11	8	3.9	0.6
Posterior tooth 3	6	11	9	4.1	1.75
Posterior tooth 4	8	12	11	4.5	2.2

All values are in mm

This bone and its ankylosed lateral teeth overlap the upper and more interior part of the bone containing the posterior tooth row. This posterior part of the PSG is narrower than the anterior part. It has four smaller, nearly horizontal teeth (Table 1, Posterior tooth 1–4) emerging at the end of the elongate bone plate with an exposed length varying between 5 mm (Posterior tooth 1) and 8 mm (Posterior tooth 4) and thickness ranging from 3.3 mm (Posterior tooth 1) to 4.5 mm (Posterior tooth 4). In contrast to the other tooth row, all tooth bases are well embedded in the jawbone.

#### Internal structures

The entire bone shows vascularisation (Fig. 4b–f), as seen in *Compagopiscis* (Rücklin et al. 2012). Although the resolution and contrast of our μCT dataset are adequate for assessing the presence and extent of gross histological structures (e.g., bone layers, or larger canals), they do not allow for a more detailed microhistological study. The teeth of both tooth rows are pointed, strongly oriented posteriorly (almost horizontal) with an upward curvature and a slight lingual orientation. In the μCT-images, the teeth show different layers of varying density. Dentinous tissue (the quality of the scan does not show enough detail to determine whether it is semidentine) surrounds the pulp cavity (Fig. 4b–f, h–i), except in Lateral tooth 1, where the pulp cavity is closed. The dorsal teeth have larger pulp cavities than the ventral ones. In some of the teeth, some vascular canals connect the pulp cavity to the vascularisation of the bone in the μCT-images (Fig. 4e).

The lateral tooth row has four teeth displaying a pulp cavity (Fig. 4c, f). The fifth, most ventral tooth has an infilled pulp cavity (Table 1, Lateral tooth 1). The pulp cavity is generally wider and longer in the younger teeth than in the older ones (Table 1). In this tooth row, two teeth show vascular canals connected to their pulp cavity (Lateral tooth 2 and 4). The quality of the scan allowed us to reconstruct four canals in Lateral tooth 2 and nine canals in Lateral tooth 4 (Appendix 1: Table 3). One of the canals in Lateral tooth 2 is partially fused to another canal. The canals are all nearly straight and originate from two spots at the base of the pulp cavity. In Lateral tooth 4, five of the nine canals are long and curved. All canals follow a posterior orientation. The posterior row of teeth comprises four teeth, all with an open pulp cavity (Fig. 4b, d, e). Posterior teeth 3 and 4 have canals preserved at the base of the pulp cavity (Appendix 1: Table 3). We reconstructed five relatively straight canals in tooth 3 and tooth 4 has one small void formed by a group of canals. The pulp cavities of these teeth also get thicker and longer towards the dorsal teeth.

#### Occlusal edge

The PSG has a rather sharp and thus potentially shearing occlusal edge located at the ventral margin (Figs. 2a and 3). Lateral tooth 1 shows signs of wear on the posterior lingual side due to the contact with the inferognathal (Fig. 3c, d). The occlusal surface does not show traces of dentinous tissue. The lingual side of the PSG has vertical depressions most noticeable at an anterior and a posterior point (Fig. 3c, d). The first furrow occurs a third of the way up between the ventral margin and the dorsal process. The second depression, double the width of the first furrow, takes place two thirds of the way up between the ventral margin and the dorsal margin. A third furrow occurs near the latter, posteriorly and together these two furrows form a line parallel to the ventral margin.

#### Remarks

The postero-lingual orientation and dorsal curvature of the teeth suggest that these could not be used for food reduction. Additionally, the occlusal edge shows no apparent contact with the teeth except for the very base of the oldest lateral tooth (Lateral tooth 1) where wear would have occurred, thus confirming their lack of feeding function. As the tooth aged, the pulp cavity was filled, meaning that the youngest teeth (Lateral tooth 5, Posterior tooth 4) have a wide pulp cavity while the pulp cavity of the oldest tooth, Lateral tooth 1, is completely infilled. The depressions on the lingual side of the PSG occur in a way that might represent traces of wear from a contact with teeth of the lower jaw (Fig. 3c, d). The shape and width of the second furrow described suggests that this might be traces left by two teeth. Taking into account that the anterior part of the inferognathal is curved, it appears plausible that the inferognathal occluded with the PSG at the ventral shearing margin and movement of the lower jaw continued into the lingual side of the PSG. This would have caused wear on the posterior lingual section of the bone. The postero-lingually directed teeth might have formed some form of dental barrier or part of a clutching mechanism that could have prevented prey from escaping the shearing action of the PSG and inferognathal. Interpretations of such function however remains speculative due to the lack of more complete fossil material, including the inferognathal and skull. Skull elements would allow the reconstructions of the mouth opening and gape along with the proper orientation of the PSG, which are important to discuss teeth function.

#### Systematic affinities and comparison

**Fig. 5:**
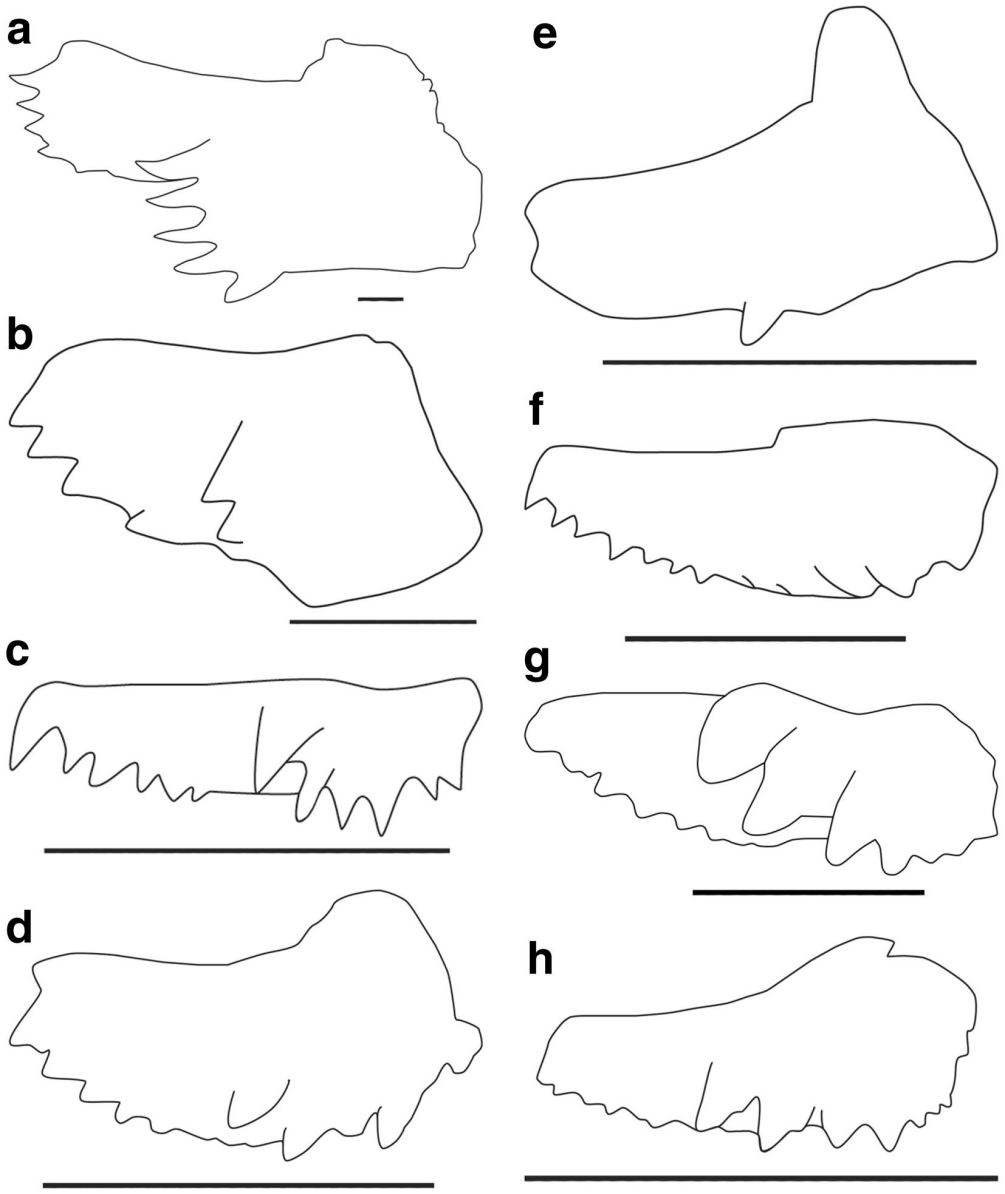
Line drawings of labial views of eubrachythoracid posterior superognathals. Note the great differences in dimensions. **a** *L. ziregensis.* **b** *Plourdosteus canadensis.* **c** *Coccosteus cuspidatus.* **d** *Harrytoombsia elegans* **e** *Mcnamaraspis kaprios.* **f** *Eastmanosteus calliaspis.* **g** *Torosteus pulchellus.* **h** *Compagopiscis croucheri*. All scale bars equal 10 mm

The overall morphology of the PSG of *L. ziregensis* gen. et sp. nov. corresponds with the condition in euarthrodires, more precisely that in eubrachythoracid arthrodires (Ørvig 1980; Smith et al. 2009; Young 2001). However, PSG elements are usually described from more or less articulated skeletons of eubrachythoracid arthrodires. Due to the attachment of these gnathal plates to the autopalatine and neurocranium via soft tissue, these elements became quickly separated taphonomically from the rest of the skeleton; by contrast, bones of the skull roof are more strongly connected at their sutures. In the following paragraphs, we compare the PSG of *L. ziregensis* gen. et sp. nov. (gen. et sp. nov. omitted subsequently for conciseness) to those of other eubrachythoracids (see Appendix 2: Table 4 for details and Fig. 5). Then, we assess whether this form is conspecific with any known eubrachythoracid from the Middle Devonian. The PSG of *Dunkleosteus* was omitted from the comparison because it does not exhibit two rows of teeth, an important character of the PSG described here.

The outline of the posterior superognathal varies in eubrachythoracids from a sub-rectangular to a more triangular form (see Table 2 and Fig. 5). The one of *L. ziregensis* is sub-rectangular like in *Plourdosteus*, *Coccosteus,* and *Mcnamaraspis* (Table 2 character 1). *L. ziregensis* has a large dorsal posterior process like all other eubrachythoracids (Table 2 character 2). However, this process is short and wide in *L. ziregensis*, similar to *Coccosteus*, *Harrytoombsia* and possibly *Plourdosteus*. This is rather different to what is described from other PSGs of eubrachythoracids like *Compagopiscis* or *Eastmanosteus*, where the dorsal process is elongate (Table 2 character 3). The PSG of *L. ziregensis* also lacks a mesial tooth row (Table 2 character 5) as in *Mcnamaraspis kaprios* and *Eastmanosteus calliaspis*. This is another character less commonly observed in posterior superognathals of brachythoracids. Additionally, *L. ziregensis* has a depression on the ventral side of the dorsal process, which is not seen in any other known eubrachythoracid PSG (Table 2 character 4).

**Table 2:** Morphological character matrix of posterior superognathals of eubrachythoracids including information on the age and locality of the material

	Age	Locality	1	2	3	4	5	6	7	8	9	Refs.
*Compagopiscis croucheri*	Frasnian	Australia	1?	1	0	0	1	1	0	0	1	Gardiner and Miles (1994)
*Coccosteus cuspidatus*	Eifelian–Givetian	UK (and other)	0	1	1	0	0–1	1	0–1	0	1	Miles and Westoll (1968)
*Plourdosteus canadensis*	Frasnian	Poland	0	1	1?	0	1	2–1	1	1	0	Ørvig (1980)
*Mcnamaraspis kaprios*	Frasnian	Australia	0	1	0	0	0	1	1	1	0	Long (1995)
*Eastmanosteus calliaspis*	Frasnian	Australia	1	1	0	0	0	1	0	0	1	Dennis-Bryan (1987)
*Harrytoombsia elegans*	Frasnian	Australia	1?	1	1	0	1	1	0	0	0	Miles and Dennis-Bryan (1979)
*Torosteus pulchellus*	Givetian–Frasnian	Australia	1?	1	0	0	1	1	0	0	1	Gardiner and Miles (1990)
*Leptodontichthys ziregensis*	Givetian	Morocco	0	1	1	1	0	2	1	1	0	This paper

Character 1 is the shape of the PSG (0: sub-rectangular, 1: triangular). Character 2 is the only character mentioned in the arthrodire character matrix of previous papers (e.g., King et al. 2016), which describes the dorsal process of the gnathal element (0: absent, 1: large, 2: small). Character 3 is the shape of the dorsal process (0: elongate, 1: short). Character 4 marks the ventral depression in the aforementioned dorsal process (0: absent, 1: present). Character 5 refers to a mesial tooth row (0: absent, 1: present). Character 6 describes tooth orientation (0: no teeth, 1: pointing downwards, 2: near parallel to the occlusion). Character 7 refers to the presence of a smooth ventral margin anterior to the lateral process (0: absent, 1: present) and character 8 anterior to the posterior process (0: absent, 1: present), both in late ontogenetic stages. Character 9 describes the height of the PSG (0: broad, 1: narrow). Uncertainties are marked by with “?”

*L. ziregensis* has teeth with a pulp cavity surrounded by dentine. The pulp cavities were gradually filled during ontogeny (Rücklin et al. 2012). The largest teeth at the end of the tooth rows have a relatively large cavity (Fig. 4d, f), suggesting that these teeth were added last. A sharp edge is developed at the ventral margin of the PSG of *L. ziregensis*. This edge does not carry teeth or unequivocal signs of wear from cutting prey items (since the surface is somewhat weathered, finer traces of wear from feeding were perhaps eroded). The presence of two rows of teeth is a common condition found among eubrachythoracids, which probably derived from gnathal elements with dental fields (Hu et al. 2019). This has been documented for derived brachythoracids, of which most date back to the Late Devonian (Smith and Johanson 2003a). Among eubrachythoracids with a documented posterior superognathal, only *Coccosteus* is of Middle Devonian age. Medium-sized specimens of *Eastmanosteus* were reported from the Middle Devonian but the only PSG assigned to this genus comes from the Late Devonian (Dennis-Bryan 1987). Thus, this makes *L. ziregensis* the first record of a morphologically derived eubrachythoracid, which is proven to have true teeth from the Middle Devonian.

The orientation of the tooth rows varies usually throughout ontogeny (Ørvig 1980). Functional teeth were likely present at the ventral margin in juveniles and entirely worn down until only the blade-like ventral edge remained, as in *Plourdosteus* (Fig. 4g). This sharp ventral margin served probably as the occlusal edge and non-functional teeth are present at its posterior end. This suggests that this specimen is an adult. Ørvig (1980) explained that teeth grew initially in a posterior direction, perpendicular to jaw occlusion, and subsequently changed their orientation to a more ventral direction. *L. ziregensis* shows teeth of various developmental stages, some of which are not parallel to the occlusal plane; they are also relatively thin, rather elongate and show a slight dorsal curvature towards the tip of all teeth, which is not seen in other arthrodires (Fig. 5).

The general shape of the PSG, tooth orientation, and the presence of a smooth margin anterior to the lateral and posterior toothed processes of *L. ziregensis* and the enigmatic *Plourdosteus canadensis* suggest that *L. ziregensis* might belong to or have a close phylogenetic relationship with plourdosteids. This is corroborated by the similarities of *L. ziregensis* with *Mcnamaraspis kaprios*, a plourdosteid with a similarly broad sub-rectangular outline of the PSG with two ventral margins preceding the lateral and posterior processes and lacking a mesial tooth like *L. ziregensis*. However, the phylogenetic position of plourdosteids is uncertain and placed with both coccosteomorphs (Ørvig 1980; Moloshnikov 2008) and pachyosteomorphs (Vézina 1990). A feasible explanation for this long-lasting debate is the potential paraphyly of the group (Carr and Hlavin 1995; Carr 2004; Anderson 2008). Besides the uncertainties of the phylogenetic position of plourdosteids, *L. ziregensis* displays a higher number of characters shared with coccosteomorphs like *Compagopiscis* or *Coccosteus*, including a rather elongate dorsal process, a broad sub-rectangular bone shape and a smooth ventral margin preceding the posterior tooth row as in *Coccosteus*, than with pachyosteomorphs like *Eastmanosteus*. However, all PSGs of eubrachythoracid placoderms, where they are known, are usually nearly one order of magnitude smaller than that of *L. ziregensis*. The longest eubrachythoracid PSG was reported from *Plourdosteus* and reaches 23.5 mm (Appendix 2: Table 4), which is still more than four times smaller than the one of *L. ziregensis*. Given the size of its PSG and compared to the data from other eubrachythoracids in this study, *L. ziregensis* was quite a large animal, possibly reaching at least 2.5–3-m long.

Nevertheless, many placoderm taxa have been described based on isolated bones or skull roofs and no information is available about their PSGs. Brachythoracids from the Middle Devonian of Morocco with no documented PSGs include *Hollardosteus* *marocanus*, a putative ?*Eastmanosteus* sp. and *Maideria falipoui*. However, *M. falipoui* is a basal brachythoracid, and its anterior superognathal (Lelièvre 1995; Johanson and Smith 2005) has a PSG-contact edge that does not fit with the posterior superognathal described here. Lehman (1976) reported *Hollardosteus* from the Late Givetian of Morocco. So far, this is the only record of this genus, suggesting it is younger than *L. ziregensis*. Lehman (1976) also hypothesized the presence of *Eastmanosteus* sp., but the affiliation of the specimen to the genus is uncertain. In addition, the PSG of different species of *Eastmanosteus* are known and even the morphologically closest one, *E. calliaspis* (Dennis-Bryan 1987) differs from that of *L. ziregensis*. *Eastmanosteus* has a different shape both of the bone and dorsal process, a vertical tooth orientation and lacks of a smooth ventral margin compared to *L. ziregensis* (Table 2).

Other Middle Devonian eubrachythoracids without PSG record have been found around the world (Appendix 3: Table 5), many of which are quite small (Sallan and Galimberti 2015). The largest eubrachythoracid from the Middle Devonian was *Carolowilhemina geognostica*, a potentially 3-m-long pachyostemorph from Spain with an unusual, elongate and cone-shaped skull (Mark-Kurik and Carls 2002). This selenosteid arthrodire has an inferognathal with no traces of teeth, unlike what is expected for the inferognathal of *L. ziregensis*, therefore rejecting a closer systematic relationship of *L. ziregensis* to that taxon. Another large eubrachythoracid is *Livosteus grandis* from the Middle Devonian of Latvia (Denison 1978). Sallan and Galimberti (2015) estimated that its body could have exceeded 2 m in length with a skull and shoulder girdle as long as 80 cm (Denison 1978). *Liv. grandis,* however, has up to 25-mm-thick bones (Denison 1978). The PSG of *L. ziregensis* is comparatively thin at 3–10 mm, and was more likely to reach 3–3.5-m long (see discussion for more), which also dismisses a potential affiliation to this species.

## Discussion

### Early and Middle Devonian forms

Very few eubrachythoracid gnathal elements have been recorded that are older than the Frasnian (Late Devonian). Some arthrodire gnathal elements have been reported from the Early Devonian of Europe (Mark-Kurik 1985; Blieck and Goujet 1991), Africa (Lelièvre 1984a), Australia (Young et al. 2001), Asia (Blieck et al. 1980), and North America (Elliott and Carr 2010). However, only two were assigned to brachythoracids (Lelièvre 1984a; Young et al. 2001) and none belong to eubrachythoracids. Among these gnathal elements, the majority has tuberculated plates. Some of these denticles are more or less arranged in rows as in the buchanosteid from Australia (Figs. 3a–g, 4a–c, 5a in Young et al. 2001; Hu et al. 2019). In other cases, these denticles fill the anterolateral face of the gnathal element as in the anterior superognathal of *Bryantolepis* (Fig. 5b in Elliott and Carr 2010). *Actinolepis spinosa* comes from Latvia and exhibits large tooth-like structures on its superognathals (Mark-Kurik 1985). However, the presence of actual dentinous tissue has yet to be tested. In addition, both superognathals are flat plates that do not possess a dorsal process. Eubrachythoracids commonly have a dorsal process on their ASG and their PSG. The presence and shape of these processes are considered to be of major importance for phylogenetic reconstruction (Carr 1991; Trinajstic and Dennis-Bryan 2009). Its absence in *Actinolepis* suggests a plesiomorphic state of the character in the taxon. The partial preservation of the PSG of *Actinolepis* does not allow the evaluation of the presence of tooth rows, although it has been previously suggested that it bears numerous teeth, possibly in rows, as in the ASG (Mark-Kurik 1985).

The Middle Devonian yielded the oldest eubrachythoracid gnathal elements, including the inferognathal of the pachyosteomorph *Squamatognathus steeprockensis* (Hanke et al. 1996b) and all three gnathal components of the Eifelian coccosteomorph *Coccosteus cuspidatus* (Miles and Westoll 1968). *Squamatognathus* has an inferognathal of ca. 24-cm long. It is toothless like most other inferognathals of this time such as the brachythoracid *Homostius* and the enigmatic arthrodire *Carolowilhelmina* (Mark-Kurik 1992; Mark-Kurik and Carls 2002). White (1952) described a posterior superognathal of an unknown brachythoracid from the Eifelian, which forms a lateral process but lacks teeth. An anterior superognathal of *Dinichthys* has been reported from the Middle Devonian of Manitoba (Whiteaves 1892). It has a lateral cusp as in other dinichthyid arthrodires (Fig. 2b in Carr 2010), but no further study was possible because the specimen has been lost (Hanke et al. 1996a). *Maideria* from the Givetian of Morocco preserves an ASG; this gnathal element is broad, the occlusal surface is covered with small pointed denticles and its anterior lateral and lateral margins present a row of teeth (Johanson and Smith 2005; Lelièvre 1995), where the histology is unknown. The late Givetian of China yielded the eubrachythoracid *Kiangyousteus*, of which a ca. 6-cm-long ASG was described (Zhu and Zhu 2013). As in *Dinichthys*, it has only one lateral cusp. An inferognathal of a dinichthyid of uncertain generic affinity (possibly *Dinichthys*) was reported from the late Givetian to early Frasnian of Iowa; it has an anterior cusp and a sharp toothless edge (Straka and Semken 1969). Thus, *L. ziregensis* represents the second oldest eubrachythoracid posterior superognathal worldwide. Additionally, given that the presence of two rows of teeth is mostly found among eubrachythoracids, it is one of the earliest superognathal elements displaying this feature with *Coccosteus*.

Among all these pre-Late Devonian gnathal elements, it has been suggested that *Actinolepis* has preserved pulp cavities (Mark-Kurik 1985). However, Young et al. (2001) later challenged this interpretation, and suggested that the spaces interpreted as remains of pulp cavities were actually preservation artifacts. The inferognathal of the Middle Devonian *Squamatognathus* presents a sculpture of semidentine on the lingual side of the element with underlying bone that is dense with a few vascular canals (Hanke et al. 1996b). The authors correlate the wear pattern and underlying bone composition with that of *Holonema westolli*. However, this is a sheet of semidentine, the sculpture does not have a tooth-shape and lacks pulp cavities. The inferognathal also possesses symphyseal denticles on the lingual angle of the terminal cusp but they appear to lack semidentine or pulp cavities. Johanson and Smith (2005) described *Maideria*’s anterior superognathal plate and noticed that one of the teeth broke off almost entirely, and thus exposes the pulp cavity. However, as Johanson and Smith (2005) also pointed out, the presence of dentine, semidentine or dentinous tissue was not verified. *L. ziregensis* has teeth composed of dentinous tissue with pulp cavities, which makes this the oldest record of teeth with evidence of these structures in eubrachythoracids and, along with *Maideria*’s pulp cavity, brachythoracids. In turn, this implies a more widespread distribution of such derived structures in the group already in the Middle Devonian.

### Taphonomy

A loose attachment of the gnathal elements to the skull explains why they are often found isolated or missing even when the skull is articulated otherwise. An isolated bone like the PSG of *Leptodontichthys* implies that the element fell off and was transported post mortem. Its reasonably good preservation, the relative completeness of rare gnathostomes findings as *Maideria* and invertebrates, such as trilobites, found in the host layer (Lelièvre 1995; Campbell et al. 2002) as well as the argillaceous-calcareous wackestone matrix surrounding the bone suggest relatively calm waters and low-energy depositional settings (Dunham 1962; Jakubowicz et al. 2019). Thus, the PSG likely did not undergo prolonged transport in a high-energy environment.

### Putative body size and position in the food web

Given the size of the posterior superognathal, *L. ziregensis* was most likely a large animal. However, estimating the size of this animal with a PSG alone is delicate, even more so when the ratio body size to PSG in arthrodires is poorly databased. For example, using the known PSG size and estimated body length of *Mcnamaraspis*, suggests *L. ziregensis* measured ca. 2.8 m. On the other hand, *L. ziregensis* reaches 5-m long when using the PSG and body length of *Eastmanosteus*. Combining the data (PSG and body lengths (for body lengths: Sallan and Galimberti 2015)) from eubrachythoracids used in the study, the mean and median suggest an estimated body length of *L. ziregensis* of 3.5 m.

Strata from the host layer contain abundant invertebrates including corals, brachiopods, crinoids, gastropods (Halamski and Baliński 2013)and articulated specimens of the world renowned huge phacopid genus *Drotops* (Hollard 1974; Struve 1995). The abundance of the latter, along with previous suggestions about the diet of arthrodires (Long 1995; Trinajstic and McNamara 1999; Anderson 2010), suggests that they might have been a food source for gnathostomes such as the placoderm *Leptodontichthys. *Furthermore, given the large size of *Leptodontichthys*, it is possible that it preyed on smaller gnathostomes, including juveniles. This concurs with the previous reports of some plourdosteids (Zakharenko 2008) and coccosteomorphs such as *Coccosteus* (Miles and Westoll 1968; Davidson and Trewin 2005) with small fish, acanthodian and juvenile placoderm remains in the stomach area or gut content. However, no direct evidence for the diet of *Leptodontichthys* is available as no stomach content is preserved, so these suggestions remain speculative. Additionally, there are no traces of food-related wear preserved on its posterior superognathal.

## Conclusion

We describe a large posterior superognathal from the Middle Devonian Drotops layer of the eastern Anti-Atlas. This gnathal element bears two rows of large teeth and because of its derived features given its time, we introduce the new taxon *Leptodontichthys ziregensis* gen. et sp. nov. and assign it to Eubrachythoraci. The morphology of the PSG and tooth orientation point towards a close relationship with the later occurring *Plourdosteus*. We thus include *L. ziregensis* in the enigmatic Plourdosteidae family. Taking the likelihood that Plourdosteidae are paraphyletic into account, *L. ziregensis* might be closely related to coccosteomorphs. However, more cranial material is required to determine its precise systematic position. The posterior superognathal of *L. ziregensis* is much bigger than the ones typical for plourdosteids and coccosteomorphs from around the world and is among the largest PSGs for Middle Devonian placoderms. The posterior orientation of the two tooth rows and the absence of teeth being oriented perpendicular to the occlusal plane is tentatively explained by an advanced age (ontogenetic stage) of the individual, where most of the functional teeth were worn down. This means that, once its functional teeth were abraded, the animal conceivably fed by crushing its food on bare bone. In addition, we also document the presence of dentinous tissue and the gradual filling of the pulp cavities in yet another ‘placoderm’. Considering the large size of the PSG, *L. ziregensis* may have been among the predators inhabiting the Maïder Basin in the eastern Anti-Atlas during the Givetian.

## Data Availability

The datasets generated and analyzed during the current study are available in the Dryad repository [doi: 10.5061/dryad.p5hqbzkng].

## Appendix 1

See Table 3.

**Table 3:** Measurements of vascular canals on each tooth of PIMUZ A/I 5022

		VC 1	VC 2	VC 3	VC 4	VC 5	VC 6	VC 7	VC 8	VC 9
Lateral tooth 2	Length	0.9	0.9	1.1	1.2	1.8				
	Width	0.2	0.3	0.3	0.3	0.2				
Lateral tooth 4	Length	0.4	0.8	0.9	1.2	1.5	1.5	2.3*	2.5*	3*
	Width	0.2	0.1	0.2	0.2	0.2	0.2	0.2	0.2	0.2
Posterior tooth 3	Length	0.8	1	1.5	1.6	2.1*				
	Width	0.3	0.2	0.3	0.2	0.2				
Posterior tooth 4	Length	0.8								
	Width	0.8								

Some of them were curved (marked by an asterisk *); the values are given without following the angles

## Appendix 2

See Table 4.

**Table 4:** Eubrachythoracid posterior superognathals used in this paper

	Specimen number	PSG length (mm)	Age	Locality	References
*Coccosteus cuspidatus*	KC6	13	Eifelian–Givetian	Scotland	Miles and Westoll (1968)
*Compagopiscis croucheri*	BMNH P50946	9	Frasnian	Australia	Gardiner and Miles (1994)
*Eastmanosteus calliaspis*	BMNH P50888	17	Frasnian	Australia	Dennis-Bryan (1987)
*Harrytoombsia elegans*	WAM704254	12	Frasnian	Australia	Miles and Dennis (1979)
*Mcnamaraspis kaprios*	WAM869676	12.5	Frasnian	Australia	Long (1995)
*Plourdosteus canadensis*	SMNH P5043	23.5	Frasnian	Poland	Ørvig (1980)
*Torosteus pulchellus*	BMNH P52553	10	Givetian–Frasnian	Australia	Gardiner and Miles (1990)
*Leptodontichthys ziregensis*	PIMUZ A/I 5022	108	Eiafelian	Morocco	This paper

## Appendix 3

See Table 5.

**Table 5:** Middle Devonian eubrachythoracids

Taxon	Group	Body size (cm)	Period	Locality	References
*Belgiosteus mortelmansi*	Coccosteomorph	36	Givetian	Belgium	Lehman (1973)
*Clarkosteus halmodeus*	Coccosteomorph	57	Givetian–Frasnian	USA (NY)	Clarke (1894)
*Coccosteus cuspidatus*	Coccosteomorph	40	Eifelian–Givetian	Scotland	Miles and Westoll (1968)
*Dickosteus threiplandi*	Coccosteomorph	50	Givetian	Scotland	Denison (1978)
*Livosteus grandis*	Coccosteomorph	210	Givetian	Latvia	Obrucheva (1962)
*Millerosteus minor*	Coccosteomorph	10	Givetian	Scotland	Denison (1978)
*Protitanichthys rockportensis*	Coccosteomorph	110	Givetian	USA (Michigan)	Case (1931)
*Watsonosteus fletti*	Coccosteomorph	58	Givetian	Scotland	Watson (1932)
*Beyrichosteus radiatus*	Coccosteomorph?	52	Givetian	Germany	Otto (2005)
*Eastmanosteus pustulosus*	Pachyosteomorph	170	Givetian–Frasnian	USA	Boylan (1973)
*?Easmanosteus sp.*	Pachyosteomorph	?	Givetian	Morocco	Lehman (1976)
*Kiangyousteus yohii*	Pachyosteomorph	120	Givetian	China	Zhu and Zhu (2013)
*Hollardosteus*	Pachyosteomorph	?	Givetian	Morocco	Lehman (1976)
*Carolowilhemina geognostica*	Pachyosteomorph	300	Eifelian	Spain	Mark-Kurik and Carls (2002)
*Panxiosteus oculus*	Pachyosteomorph	38	Givetian–Frasnian	China	Wang (1979)
*Eastmanosteus yunnanensis*	Pachyosteomorph	110	Givetian	China	Wang (1991)

The body sizes in red are estimates taken from Sallan and Galimberti (2015). However, the estimate for *Belgiosteus* appears erroneous (estimated at 3.6-m long with a 62-mm cranial roof). Considering the cranium sizes given by Denison (1978), *Belgiosteus* was a little smaller that *Coccosteus*, which suggests that the body size written by Sallan and Galimberti may have resulted from a misplaced comma with 360 mm (0.36 m) being correct. Values marked by “?” are unknown

## Abbreviations

ASG: Anterior superognathal
PSG: Posterior superognathal
PIMUZ: Paläontologisches Intstitut und Museum der Universität Zürich, Switzerland

## References

[CR1] Anderson, P. S. L. (2008). Shape variation between arthrodire morphotypes indicates possible feeding niches. *Journal of Vertebrate Paleontology,**28,* 961–969. 10.1671/0272-4634-28.4.961.

[CR2] Anderson, P. S. L. (2010). Using linkage models to explore skull kinematic diversity and functional convergence in arthrodire placoderms. *Journal of Morphology*. 10.1002/jmor.10850. 10.1002/jmor.10850 20623651

[CR3] Anderson, P. S. L., & Westneat, M. W. (2006). Feeding mechanics and bite force modelling of the skull of *Dunkleosteus terrelli*, an ancient apex predator. *Biology Letters,**3,* 77–80. 10.1098/rsbl.2006.0569. 10.1098/rsbl.2006.0569 PMC2373817 17443970

[CR4] Blieck, A., Golshani, F., Goujet, D., Hamdi, A., Janvier, P., Mark-Kurik, E., & Martin, M. (1980). A new vertebrate locality in the Eifelian of the Khush-Yeilagh Formation, Eastern Alborz. *Iran. Palaeovertebrata,**9,* 133–154.

[CR5] Blieck, A., & Goujet, D. (1991). Les vertébrés du Dévonien inférieur d’Arville et de Nonceveux (Belgique). *Annales de la Société géologique du Nord,**1,* 67–78.

[CR88] Boylan, J. C. (1973). *Eastmanosteus, A Placoderm from the Devonian of North America*. Unpublished PhD dissertation, Columbia University, 523 pp.

[CR7] Brazeau, M. D. (2009). The braincase and jaws of a Devonian ‘acanthodian’ and modern gnathostome origins. *Nature,**457,* 305–308. 10.1038/nature07436. 19148098 10.1038/nature07436

[CR8] Brazeau, M. D., & Friedman, M. (2015). The origin and early phylogenetic history of jawed vertebrates. *Nature,**520,* 490–497. 10.1038/nature14438. 25903631 10.1038/nature14438 PMC4648279

[CR9] Brazeau, M. D., Giles, S., Dearden, R. P., Jerve, A., Ariunchimeg, Y., Zorig, E., et al. (2020). Endochondral bone in an Early Devonian ‘placoderm’ from Mongolia. *Nature Ecology & Evolution*. 10.1038/s41559-020-01290-2. 10.1038/s41559-020-01290-2 32895518

[CR10] Burrow, C. J. (2003). Comment on “Separate Evolutionary Origins of Teeth from Evidence in Fossil Jawed Vertebrates.” *Science*. 10.1126/science.1083877. 10.1126/science.300.5626.1661a 12805521

[CR11] Campbell, K., Barwick, R., Chatterton, B., & Smithson, T. (2002). A new Middle Devonian dipnoan from Morocco: structure and histology of the dental plates. *Records of the Western Australian Museum,**21,* 39–61. 10.18195/issn.0312-3162.21(1).2002.039-061.

[CR12] Carr, R. K. (1991). Reanalysis of Heintzichthys gouldii (Newberry), an aspinothoracid arthrodire (Placodermi) from the Famennian of northern Ohio, with a review of brachythoracid systematics. *Zoological Journal of the Linnean Society,**103,* 349–390. 10.1111/j.1096-3642.1991.tb00909.x.

[CR13] Carr, R. K. (2004). Recognizing paraphyletic stem groups: A case study in the analysis of eubrachythoracid arthrodires (Placodermi). In G. Arratia, M. V. H. Wilson, & R. Cloutier (Eds.), *Recent advances in the origin and early radiation of vertebrates*. Germany: Verlag Dr. Friedrich Pfeil, München.

[CR14] Carr, R. (2010). Paleoecology of *Dunkleosteus terrelli* (Placodermi: Arthrodira). *Kirtlandia,**57,* 36–45.

[CR15] Carr, R. K., & Hlavin, W. J. (1995). Dinichthyidae (Placodermi): A paleontological fiction? *Geobios,**28,* 85–87. 10.1016/s0016-6995(95)80092-1.

[CR89] Case, E. C. (1931). Arthrodiran remains from the Devonian of Michigan. *Contributions for the Museum of Paleontology, University of Michigan*, *3*, 163–182.

[CR98] Clarke J. M. (1894). New or rare species of fossils from the horizons of the Livonia salt shaft. Report State Geological Survey of New York, pp. 162–168.

[CR16] Coatham, S. J., Vinther, J., Rayfield, E. J., & Klug, C. (2020). Was the Devonian placoderm *Titanichthys* a suspension feeder? *Royal Society Open Science,**7,* 200272. 10.1098/rsos.200272. 32537223 10.1098/rsos.200272 PMC7277245

[CR17] Davidson, R. G., & Trewin, N. H. (2005). Unusual preservation of the internal organs of acanthodian and actinopterygian fish in the Middle Devonian of Scotland. *Scottish Journal of Geology,**41,* 129–134. 10.1144/sjg41020129.

[CR18] Davies, T. G., Rahman, I. A., Lautenschlager, S., Cunningham, J. A., Asher, R. J., Barrett, P. M., et al. (2017). Open data and digital morphology. *Proceedings of the Royal Society Biological Sciences*. 10.1098/rspb.2017.0194. 10.1098/rspb.2017.0194 PMC5394671 28404779

[CR19] Denison, R. (1978). *Handbook of Paleoichthyology* (Vol. 2). Gustav Fischer, Stuttgart: Placodermi.

[CR20] Dennis, K., & Miles, R. S. (1980). New durophagous arthrodires from Gogo, Western Australia. *Zoological Journal of the Linnean Society,**69,* 43–85. 10.1111/j.1096-3642.1980.tb01932.x.

[CR21] Dennis-Bryan, K. (1987). A new species of eastmanosteid arthrodire (Pisces: Placodermi) from Gogo, Western Australia. *Zoological Journal of the Linnean Society,**90,* 1–64. 10.1111/j.1096-3642.1987.tb01347.x.

[CR22] Dennis-Bryan, K., & Miles, R. S. (1983). Further eubrachythoracid arthrodires from Gogo, Western Australia. *Zoological Journal of the Linnean Society,**77,* 145–173. 10.1111/j.1096-3642.1983.tb00527.x.

[CR23] Donoghue, P. C. J., & Rücklin, M. (2014). The ins and outs of the evolutionary origin of teeth. *Evolution & Development,**18,* 19–30. 10.1111/ede.12099. 25219878 10.1111/ede.12099

[CR24] Dunham, R. (1962). Classification of carbonate rocks according to depositional textures. Classification of Carbonate Rocks—A Symposium 108–121.

[CR25] Elliott, D. K., & Carr, R. K. (2010). A new species of *Bryantolepis* Camp, Welles, and Green, 1949 (Placodermi, Arthrodira) from the Early Devonian Water Canyon Formation of northern Utah and southern Idaho, with comments on the endocranium. *Kirtlandia,**57,* 22–35.

[CR26] Frey, L., Coates, M. I., Tietjen, K., Rücklin, M., Klug, C. (2020). A new symmoriiform from the Late Devonian of Morocco: novel jaw function in ancient sharks. Communications Biology (in press). 10.1038/s42003-020-01394-2 PMC7672094 33203942

[CR27] Frey, L., Rücklin, M., Korn, D., & Klug, C. (2018). Late Devonian and Early Carboniferous alpha diversity, ecospace occupation, vertebrate assemblages and bio-events of southeastern Morocco. *Palaeogeography, Palaeoclimatology, Palaeoecology,**496,* 1–17. 10.1016/j.palaeo.2017.12.028.

[CR28] Gardiner, B. G., & Miles, R. S. (1990). A new genus of eubrachythoracid arthrodire from Gogo, Western Australia. *Zoological Journal of the Linnean Society,**99,* 159–204. 10.1111/j.1096-3642.1990.tb00566.x.

[CR29] Gardiner, B. G., & Miles, R. S. (1994). Eubrachythoracid arthrodires from Gogo, Western Australia. *Zoological Journal of the Linnean Society,**112,* 443–477. 10.1111/j.1096-3642.1994.tb00331.x.

[CR30] Halamski, A., & Baliński, A. (2013). Middle Devonian brachiopods from the southern Maïder (eastern Anti-Atlas, Morocco). *Annales Societatis Geologorum Poloniae,**83,* 243–307.

[CR31] Hanke, G. F., Stewart, K. W., & Lammers, G. E. (1996a). *Eastmanosteus lundarensis* sp. nov. from the Middle Devonian Elm Point and Winnipegosis Formations of Manitoba. *Journal of Vertebrate Paleontology,**16,* 606–616. 10.1080/02724634.1996.10011351.

[CR32] Hanke, G. F., Stewart, K. W., & Lammers, G. E. (1996b). *Squamatognathus steeprockensis* gen. et sp. nov., an arthrodire inferognathal from the Middle Devonian Elm Point Formation of Manitoba. *Journal of Vertebrate Paleontology,**16,* 617–622. 10.1080/02724634.1996.10011352.

[CR33] Hollard, H. (1974). Recherches sur la stratigraphie des formations du Dévonien Moyen, de l’Emsien supérieur, au Frasnien, dans le sud du Tafilalt et dans le Ma’der (Anti-Atlas oriental). *Notes du Service géologique du Maroc,**36,* 7–68.

[CR34] Hu, Y.-Z., Young, G., Burrow, C., Zhu, Y.-A., & Lu, J. (2019). High resolution XCT scanning reveals complex morphology of gnathal elements in an Early Devonian arthrodire. *Palaeoworld,**28,* 525–534. 10.1016/j.palwor.2018.12.003.

[CR35] Huysseune, A., & Witten, P. E. (2008). An evolutionary view on tooth development and replacement in wild Atlantic salmon (*Salmo salar* L.). *Evolution & Development,**10,* 6–14. 10.1111/j.1525-142x.2007.00209.x. 18184353 10.1111/j.1525-142X.2007.00209.x

[CR36] Jakubowicz, M., Król, J., Zapalski, M. K., Wrzołek, T., Wolniewicz, P., & Berkowski, B. (2019). At the southern limits of the Devonian reef zone: Palaeoecology of the Aferdou el Mrakib reef (Givetian, eastern Anti-Atlas, Morocco). *Geological Journal,**54,* 10–38. 10.1002/gj.3152.

[CR37] Johanson, Z., & Smith, M. M. (2005). Origin and evolution of gnathostome dentitions: a question of teeth and pharyngeal denticles in placoderms. *Biological Reviews,**80,* 303–345. 10.1017/s1464793104006682. 15921053 10.1017/s1464793104006682

[CR38] Kaufmann, B. (1998). Facies, stratigraphy and diagenesis of Middle Devonian reef- and mud-mounds in the Mader (eastern Anti-Atlas, Morocco). *Acta Geologica Polonica,**48,* 43–106.

[CR39] King, B., Qiao, T., Lee, M. S. Y., Zhu, M., & Long, J. A. (2016). Bayesian Morphological Clock Methods Resurrect Placoderm Monophyly and Reveal Rapid Early Evolution in Jawed Vertebrates. *Systematic Biology,**66,* 499–516. 10.1093/sysbio/syw107. 10.1093/sysbio/syw107 27920231

[CR40] Lehman, J. P. (1956). Les Arthrodires du Dévonien supérieur du Tafilalt (Sud Marocain). *Notes et Mémoires du Service Géologique du Maroc,**129,* 1–70.

[CR41] Lehman, J. P. (1964). A propos de quelques Arthrodires et Ichthyodorulites sahariens. *Mémoire IFAN,**68,* 193–200.

[CR90] Lehman, J. P. (1973). Un Nouveau coccostéomorphe, Belgiosteus mortelmansi. *Annales de paléontologie*, *59*, 3–14.

[CR42] Lehman, J. P. (1976). Nouveaux poissons fossiles du Dévonien du Maroc. *Annales de Paléontologie Vertébrés,**62,* 1–34.

[CR43] Lelièvre, H. (1984a). *Antineosteus lehmani* n. g., n. sp., nouveau Brachythoraci du Dévonien inférieur du Maroc présaharien. Remarques sur la paléobiogéographie des Homostéides de l’Emsien. *Annales de Paléontologie,**70,* 115–158.

[CR44] Lelièvre, H. (1984b). *Atlantidosteus hollardi* n. g., n. sp., nouveau Brachythoraci (Vertébrés, Placodermes) du Dévonien inférieur du Maroc présaharien. *Bulletin du Muséum national d’histoire naturelle,**6,* 197–208.

[CR45] Lelièvre, H. (1995). Description of *Maideria falipoui* n. g., n. sp., a long snouted brachythoracid (Vertebrata, Placodermi, Arthrodira) from the Givetian of Maider (South Morocco), with a phylogenetic analysis of primitive brachythoracids. *Bulletin du Muséum national d’histoire naturelle,**17,* 163–207.

[CR46] Long, J. A. (1995). A new plourdosteid arthrodire from the Upper Devonian Gogo Formation of Western Australia. *Palaeontology,**38,* 39–62.

[CR47] Long, J. A., & Trinajstic, K. (2010). The Late Devonian Gogo Formation Lägerstatte of Western Australia: Exceptional Early Vertebrate Preservation and Diversity. *Annual Review of Earth and Planetary Sciences,**38,* 255–279. 10.1146/annurev-earth-040809-152416.

[CR48] Long, J. A., Trinajstic, K., & Johanson, Z. (2009). Devonian arthrodire embryos and the origin of internal fertilization in vertebrates. *Nature,**457,* 1124–1127. 10.1038/nature07732. 19242474 10.1038/nature07732

[CR49] Long, J. A., Trinajstic, K., Young, G. C., & Senden, T. (2008). Live birth in the Devonian period. *Nature,**453,* 650–652. 10.1038/nature06966. 18509443 10.1038/nature06966

[CR50] Mark-Kurik, E. (1985). *Actinolepis spinosa* n. sp. (Arthrodira) from the Early Devonian of Latvia. *Journal of Vertebrate Paleontology,**5,* 287–292. 10.1080/02724634.1985.10011866.

[CR51] Mark-Kurik, E. (1992). The inferognathal in the Middle Devonian arthrodire *Homostius*. *Lethaia,**25,* 173–178. 10.1111/j.1502-3931.1992.tb01382.x.

[CR52] Mark-Kurik, E., & Carls, P. (2002). A long-snouted Late Eifelian arthrodire from Aragón (Spain). *Revista Española de Paleontología,**17,* 117–135.

[CR53] Miles, R. S. (1969). Features of Placoderm Diversification and the Evolution of the Arthrodire Feeding Mechanism. *Transactions of the Royal Society of Edinburgh,**68,* 123–170. 10.1017/s0080456800014629.

[CR54] Miles, R. S. (1971). The Holonematidae (placoderm fishes), a review based on new specimens of Holonema from the Upper Devonian of Western Australia. *Philosophical Transactions of the Royal Society of London B, Biological Sciences,**263,* 101–234. 10.1098/rstb.1971.0111.

[CR55] Miles, R. S., & Dennis, K. (1979). A primitive eubrachythoracid arthrodire from Gogo, Western Australia. *Zoological Journal of the Linnean Society,**66,* 31–62. 10.1111/j.1096-3642.1979.tb01900.x.

[CR56] Miles, R. S., & Westoll, T. S. (1968). IX.—The Placoderm Fish Coccosteus cuspidatus Miller ex Agassiz from the Middle Old Red Sandstone of Scotland. Part I. Descriptive Morphology. *Transactions of the Royal Society of Edinburgh,**67,* 373–476. 10.1017/s0080456800024078.

[CR57] Moloshnikov, S. V. (2008). The placoderm *Plourdosteus livonicus* (Eastman) in the early Frasnian of the Central Devonian Field and the trophic structure of the Mikhailovskii Fish Assemblage. *Paleontological Journal,**42,* 607–614. 10.1134/s0031030108060063.

[CR91] Obrucheva, O. P. (1962). *Pantsirnye ryby Devona SSSR (Kokkosteidy i Dinikhtiidy)*. Moskow: Izdatel'stvo Moskovskogo universiteta.

[CR58] Ørvig, T. (1980). Histologic studies of Ostracoderms, Placoderms and Fossil Elasmobranchs. *Zoologica Scripta,**9,* 219–239. 10.1111/j.1463-6409.1980.tb00665.x.

[CR92] Otto, M. (2005). Beyrichosteus radiatus n. g., n. sp., a brachythoracid arthrodire with completely ossified endocranium from the Middle Devonian (Givetian) of the Eifel hills. *Paläontologische Zeitschrift*, *79*, 493–505.

[CR93] Rixon, A. E. (1976). *Fossil animal remains: their preparation and conservation*. London: Athlone Press.

[CR59] Rücklin, M. (2011). First selenosteid placoderms from the eastern Anti-Atlas of Morocco; osteology, phylogeny and palaeogeographical implications. *Palaeontology,**54,* 25–62. 10.1111/j.1475-4983.2010.01026.x.

[CR60] Rücklin, M., & Clément, G. (2017). Une revue des placodermes et sarcoptérygiens du Dévonien du Maroc. Zhouri S. eds. Paléontologie des vertébrés du Maroc: état des connaissances. Mémoires de la Société Géologique de France, 2017, t. 180. 79–101.

[CR61] Rücklin, M., Donoghue, P. C. J., Cunningham, J. A., Marone, F., & Stampanoni, M. (2014). Developmental paleobiology of the vertebrate skeleton. *Journal of Paleontology,**88,* 676–683. 10.1666/13-107. 26306050 10.1666/13-107 PMC4545513

[CR62] Rücklin, M., Donoghue, P. C. J., Johanson, Z., Trinajstic, K., Marone, F., & Stampanoni, M. (2012). Development of teeth and jaws in the earliest jawed vertebrates. *Nature,**491,* 748–751. 10.1038/nature11555. 23075852 10.1038/nature11555

[CR63] Sallan, L., & Galimberti, A. K. (2015). Body-size reduction in vertebrates following the end-Devonian mass extinction. *Science,**350,* 812–815. 10.1126/science.aac7373. 26564854 10.1126/science.aac7373

[CR64] Smith, M. M., Fraser, G. J., & Mitsiadis, T. A. (2009). Dental lamina as source of odontogenic stem cells: evolutionary origins and developmental control of tooth generation in gnathostomes. *Journal of Experimental Zoology Part B: Molecular and Developmental Evolution,**312B,* 260–280. 10.1002/jez.b.21272. 19156674 10.1002/jez.b.21272

[CR65] Smith, M. M., & Johanson, Z. (2003a). Separate evolutionary origins of teeth from evidence in fossil jawed vertebrates. *Science,**299,* 1235–1236. 10.1126/science.1079623. 12595693 10.1126/science.1079623

[CR66] Smith, M. M., & Johanson, Z. (2003b). Response to comment on “separate evolutionary origins of teeth from evidence in fossil jawed vertebrates.” *Science,**300,* 1661c–11661. 10.1126/science.1084686. 12595693 10.1126/science.1079623

[CR67] Struve, W. (1995). Beiträge zur Kenntnis der Phacopina (Trilobita), 18: Die Riesen-Phacopiden aus dem Ma’der, SEmarokkanische Prä-Sahara. *Senckenbergiana Lethaea,**75,* 77–129.

[CR94] Straka, J. J., & Semken, H. A. (1969). A dinichthyid in Middle Devonian of Iowa. *Journal of Paleontology*, *43*, 1423–1428.

[CR95] Toombs, H. A., & Rixon, A. E. (1959). The use of acids in the preparation of vertebrate fossils. *Curator*, *2*, 304–312.

[CR68] Trinajstic, K., Boisvert, C., Long, J., Maksimenko, A., & Johanson, Z. (2015). Pelvic and reproductive structures in placoderms (stem gnathostomes). *Biological Reviews,**90,* 467–501. 10.1111/brv.12118. 24889865 10.1111/brv.12118

[CR69] Trinajstic, K., & Dennis-Bryan, K. (2009). Phenotypic plasticity, polymorphism and phylogeny within placoderms. *Acta Zoologica,**90,* 83–102. 10.1111/j.1463-6395.2008.00363.x.

[CR70] Trinajstic, K., & McNamara, K. J. (1999). Heterochrony and phylogenetic trends. *Records of the Western Australian Museum,**57,* 93–106.

[CR71] Trinajstic, K., & Roelofs, B. (2018). Placoderm Life Histories. *Encyclopedia of Animal Cognition and Behavior*. 10.1007/978-3-319-47829-6_1162-1.

[CR72] Vandenplas, S., Clercq, A. D., & Huysseune, A. (2014). Tooth replacement without a dental lamina: The search for epithelial stem cells in *Polypterus senegalus*. *Journal of Experimental Zoology Part B: Molecular and Developmental Evolution,**322,* 281–293. 10.1002/jez.b.22577. 24890316 10.1002/jez.b.22577

[CR73] Vaškaninová, V., Chen, D., Tafforeau, P., Johanson, Z., Ekrt, B., Blom, H., & Ahlberg, P. E. (2020). Marginal dentition and multiple dermal jawbones as the ancestral condition of jawed vertebrates. *Science,**369,* 211–216. 10.1126/science.aaz9431. 32647004 10.1126/science.aaz9431

[CR74] Vaškaninová, V., & Kraft, P. (2014). The largest Lower Devonian placoderm—*Antineosteus rufus* sp. nov. from the Barrandian area (Czech Republic). *Bulletin of Geosciences*. 10.3140/bull.geosci.1450.

[CR75] Vézina, D. (1990). Les Plourdosteidae fam. nov. (Placodermi, Arthrodira) et leurs relations phylétiques au sein des Brachythoraci. *Canadian Journal of Earth Sciences,**27,* 677–683. 10.1139/e90-065.

[CR96] Wang, C.-C. (1979). A new family of Arthrodira from Yunnan, China. *Vertebrata PalAsiatica*, *17*, 179–188.

[CR76] Wang, J. Q. (1991). The Antiarchi from early Silurian of Hunan. *Vertebrata PalAsiatica,**21,* 240–244.

[CR97] Watson, D. M. S. (1932). On three new species of fish from the Old Red Sandstone of Orkney and Shetland. *Summary of Progress of the Geological Survey*, *1931*, 157–166.

[CR77] Wendt, J. (1985). Disintegration of the continental margin of northwestern Gondwana: Late Devonian of the eastern Anti-Atlas (Morocco). *Geology,**13,* 815. 10.1130/0091-7613(1985)13%3c815:dotcmo%3e2.0.co;2.

[CR78] White, E. (1952). Australian arthrodires. *Bulletin of the British Museum (Natural History) Geology,**1,* 249–304.

[CR79] Whiteaves, J. F. (1892). The fossils of the Devonian rocks of the islands, shores or immediate vicinity of lakes Manitoba and Winnipegosis, Contributions to Canadian Paleontology. *Geological Survey of Canada,**1,* 1–359. 10.4095/225768.

[CR80] Young, G. C. (2003a). Did placoderm fish have teeth? *Journal of Vertebrate Paleontology,**23,* 987–990.

[CR81] Young, G. C. (2003b). A new species of *Atlantidosteus* Lelièvre, 1984 (Placodermi, Arthrodira, Brachythoraci) from the Middle Devonian of the Broken River area (Queensland, Australia). *Geodiversitas,**25,* 681–694.

[CR82] Young, G. C. (2010). Placoderms (Armored Fish): Dominant Vertebrates of the Devonian Period. *Annual Review of Earth and Planetary Sciences,**38,* 523–550. 10.1146/annurev-earth-040809-152507.

[CR83] Young, G. C., Lelièvre, H., & Goujet, D. (2001). Primitive jaw structure in an articulated brachythoracid arthrodire (placoderm fish; early Devonian) from southeastern Australia. *Journal of Vertebrate Paleontology,**21,* 670–678. 10.1671/0272-4634(2001)021[0670:pjsiaa]2.0.co;2.

[CR84] Zakharenko, G. V. (2008). Possible evidence of predation in placoderms (Pisces) of the Evlanovo Basin of Central Russia. *Palaeontological Journal,**42,* 522–525.

[CR85] Zhu, M., Ahlberg, P. E., Pan, Z., Zhu, Y., Qiao, T., Zhao, W., et al. (2016). A Silurian maxillate placoderm illuminates jaw evolution. *Science,**354,* 334–336. 10.1126/science.aah3764. 27846567 10.1126/science.aah3764

[CR86] Zhu, M., Yu, X., Ahlberg, P. E., Choo, B., Lu, J., Qiao, T., et al. (2013). A Silurian placoderm with osteichthyan-like marginal jaw bones. *Nature,**502,* 188–193. 10.1038/nature12617. 24067611 10.1038/nature12617

[CR87] Zhu, Y.-A., & Zhu, M. (2013). A redescription of *Kiangyousteus yohii* (Arthrodira: Eubrachythoraci) from the Middle Devonian of China, with remarks on the systematics of the Eubrachythoraci. *Zoological Journal of the Linnean Society,**169,* 798–819. 10.1111/zoj.12089.

